# Temperature and Host Plant Impacts on the Development of *Spodoptera litura* (Fabricius) (Lepidoptera: Noctuidae): Linear and Nonlinear Modeling

**DOI:** 10.3390/insects14050412

**Published:** 2023-04-26

**Authors:** Rameswor Maharjan, Seoyeon Hong, Jeongjoon Ahn, Youngnam Yoon, Yunwoo Jang, Jungin Kim, Myounghee Lee, Kido Park, Hwijong Yi

**Affiliations:** 1 Crop Production Technology Research Division, Department of Southern Area Crop Science, National Institute of Crop Science, Rural Development Administration, Miryang 50424, Republic of Korea; mrameswor@korea.kr (R.M.); agriculture63@korea.kr (S.H.); yoonyn@korea.kr (Y.Y.); ywj2012@korea.kr (Y.J.); pkd@korea.kr (K.P.); 2 Research Institute of Climate Change and Agriculture, National Institute of Crop Science, Rural Development Administration, Jeju 63240, Republic of Korea; j2ahn33@korea.kr; 3 Upland Crop Breeding Research Division, Department of Southern Area Crop Science, National Institute of Crop Science, Rural Development Administration, Miryang 50424, Republic of Korea; kji1204@korea.kr (J.K.); emhee@korea.kr (M.L.)

**Keywords:** tobacco cutworm, mathematical models, nutrient contents, spring emergence, forecasting, management

## Abstract

**Simple Summary:**

The tobacco cutworm, *Spodoptera litura* (Fabricius) (Lepidoptera: Noctuidae), is a highly polyphagous and destructive insect pest, damaging a wide range of vegetables and field crops in Korea. Timely management is very important, as delays can lead to considerable economic losses. For timely management, the development of precise forecasting models is important in pest management. In this study, we estimated the temperature-based thermal requirements of *S. litura* with linear and nonlinear models on host plants to develop models to foresee the spring emergence and population dynamics of *S. litura*. We found a significant impact of temperature and host plant on the development of *S. litura*. Development was found to be inversely proportionate to temperature, despite no development at extreme temperatures. Based on the estimated thermal requirements, we developed a forecasting model, and this predicated the spring emergence of *S. litura* in May–June. Thus, the continuous observation of *S. litura* in crop fields is required in order to prepare more advanced administration measures against *S. litura*.

**Abstract:**

The tobacco cutworm, *Spodoptera litura* (Fabricius) (Lepidoptera: Noctuidae), is one of the most serious pests in field crops, vegetables, and ornamentals. Temperatures (15, 20, 25, 27, 30, 35, and 40 °C), host plants (soybean (*Glycine max* (L.)), maize (*Zea mays* L.), groundnut (*Arachis hypogaea* L.) and azuki bean (*Vigna angularis* (Willd.) Ohwi & H. Ohashi,), and the artificial diet-dependent developmental parameters and survival of *S. litura* were examined in this study. Stage-specific parameters such as threshold development temperature (LDT) and thermal constant (K) (Degree day (DD)) were determined by linear and nonlinear models (Sharpe–Schoolfield–Ikemoto), respectively. The total developmental time (egg–adult) decreased with increasing temperature on host plants and with an artificial diet. The total immature developmental time varied from 106.29, 107.57, 130.40, 111.82, and 103.66 days at 15 °C to 22.47, 21.25, 25.31, 18.30, and 22.50 days at 35 °C on soybean, maize, groundnut, azuki bean, and artificial diet, respectively. The LDT for the total immature completion was 7.50, 9.48, 11.44, 12.32, and 7.95 °C on soybean, maize, groundnut, azuki bean, and artificial diet, respectively. The K for the total immature completion was 587.88, 536.84, 517.45, 419.44, and 586.95 DD on soybean, maize, groundnut, azuki bean, and artificial diet, respectively. Temperature and host plant interaction also influenced the longevity and survival of adults. The findings of this study can be used to predict the number of generations, spring emergence, and population dynamics of *S. litura*. The nutrient content analysis of the host plants is discussed in terms of the developmental patterns of *S. litura*.

## 1. Introduction

The tobacco cutworm, *Spodoptera litura* (Fabricius) (Lepidoptera: Noctuidae), is an economic polyphagous pest that damages a wide range of crop, vegetable, and ornamental hosts in temperate and sub-tropical regions of Asia, Australasia, and the Pacific Islands [1,2,3,4]. It is known to attack 112 cultivated plant species in different parts of the world [5,6,7], with approximately 60 species being found in India [2]. Plant damage from larvae is the most serious problem due to the heavy feeding nature and insecticide resistance of the cutworm [8] and the lack of effective controls [9,10,11]. It can cause significant defoliation damage ranging anywhere between 26% and 100% due to vigorous defoliation [12], and sometimes up to 100% in field conditions [13]. Due to the severe damage it causes, *S. litura* infestations lead to considerable crop and economic losses [14,15,16,17,18,19]. The severe damage caused by this species has resulted in a 100% groundnut yield loss [14]. In Korea, *S. litura* is widespread in almost all provinces and inflicts significant damage to various crops and vegetables [20,21,22]. Further, there is a significant threat of a potential distribution expansion of *S. litura* on the Korean peninsula, as the species is found in a hot spot for climate change, especially global warming [22].

The nutritional quality of host plants varies according to each plant’s characteristics. Host plants offer diverse levels of nutritional content for different insect species, and they can have considerable effects on the life histories of these insects [23,24,25]. Hence, identifying the host-plant-mediated life table parameters of *S. litura* would provide fundamental information on the most damaging stages and help to measure and understand the population growth capacity of the species under specified conditions [26]. A deeper understanding of the host plant-based biology of insects will also provide information on the host suitability of herbivore insect species. Several previous studies reported the effects of different host plants on the biological parameters of *S. litura* under different environmental conditions [5,13,27,28,29,30,31,32,33,34]. However, the aforementioned studies focused on different host plants to those studied in this paper regarding the development, survival, pupal weight, and oviposition of *S. litura* under the same environmental conditions, and none of the prior research studied the interactive effects of constant temperature and host plants on the development of *S. litura*.

Insects are physiologically sensitive to external environmental factors due to being poikilothermic organisms; environmental factors govern the developmental rate and geographical distribution of insects, and temperature is considered one of the more significant factors affecting the overall developmental processes of insect species [35,36,37]. Pest forecasting, management, and pest risk analysis all depend on having access to information about a pest’s development, survival, and reproduction under various environmental conditions. In particular, temperature has a significant impact on the development, population dynamics, distribution, and survival of insect species [38,39,40,41,42], as well as on biological characteristics such as sex ratio [43], adult longevity, survival, and oviposition [44,45,46]. Insect development is very sensitive to temperature changes, and even slight variations in temperature can lead to huge differences in insect phenology [41,42,47]. Predicting the seasonal occurrence and abundance of any pest is essential for the accurate scheduling of control tactics. Such predictions require an understanding of the relationship between the insect development rate and temperature, especially with regard to prospective climate change [48,49], and this relationship is often described in temperature-driven phenology models based on the degree-day [50]. Although there are some studies related to the diet–temperature-mediated development of *S. litura* in Korea [22,51], there have been no studies examining the interactive effects of temperature and host plants on the developmental phenology and distribution of *S. litura* on host plants in Korea. This study examined the influence of temperature, host plant, and their interactions on the development of *S. litura*. Furthermore, this study developed a predictive model of pest distribution by estimating thermal requirements of *S. litura* using a climate change framework.

## 2. Materials and Methods

### 2.1. Insect Collection and Rearing

The larvae of *S. litura* were collected from a soybean field in the National Institute of Crop Science, Rural Development Administration (Miryang, Gyeongsangnam Province; 35°49′40″ N, 128°74′01″ E, Republic of Korea) in 2020 and reared on an artificial diet [52]. Newly emerged adults were kept separately inside acryl cages (40 × 40 × 40 cm, with side ventilation) along with greenhouse-grown soybean plants, and adults were fed a solution of honey (10%) and water. All of the cages were maintained under laboratory conditions (26 ± 1 °C, 60 ± 5% Relative Humidity (RH), and a 16:8 h L:D photoperiod). For the experiment, soybean leaves with egg clusters attached were collected by clipping the mother plant, and the egg clusters attached to the leaves were used in the experiment.

### 2.2. Host Food

The host plants tested in the experiment were selected based on their economic value and for their known associations with *S. litura* in Korea. The four host plants consisted of three leguminous and one cereal species: soybean (*Glycine max* (L.) Merr.; cv. Daewon; Fabaceae), groundnut (*Arachis hypogaea* L.; cv. Sinpalkwong; Fabaceae) and azuki bean (*Vigna angularis* (Willd.) Ohwi & H. Ohashi; cv. Hongeon; Fabaceae), maize (*Zea mays* L.; cv. Hangkeummaschal; Poaceae), and an artificial diet. The artificial diet used in this experiment is composed of several nutritional ingredients required for normal development, and was chosen given its use in other assays of defoliating insects [52,53,54], and at least to see the developmental pattern and closeness of the insects’ performance on artificial and natural foods. The seeds of all host plants were planted separately in plastic pots (18 cm dia. × 13 cm height) in commercial soil media (Baroker, Seoul Bio., Seoul, Republic of Korea) without any pesticides in a greenhouse at 28 ± 1 °C, 60–80% RH, and in a natural photoperiod. Approximately 2–3 week-old leaves of each host plant were used in the experiment. In addition, being scientifically accepted and a widely used medium for rearing of several insect species, the artificial diet [52] was also used in this experiment.

### 2.3. Experimental Procedure

The experiment began by exposing adults (<5 d old, reared on artificial diet) to greenhouse-grown soybean plants (cv. Poonsannamool; 10–12 leaves/plant; 2 weeks old without pesticides) for 24–48 h to obtain eggs. Exposure was performed in acryl cages (40 × 40 × 40 cm, with side ventilation), maintained at 26 ± 1 °C, 60 ± 5% RH, and a 16:8 h L:D photoperiod, and adults were fed a honey (10%) and water solution. Eggs were obtained 2 days after exposure to adults for the experiment. Once oviposition occurred on soybean leaves, a single egg cluster (non-overlapping) was separated and counted. Egg viability was determined based on the shape, size, and color of eggs using a microscope (LEICA EZ4D, Wetzlar, Germany) [55]. The selected egg clusters attached to the soybean leaves were placed into a Petri dish (9.8 cm dia. × 4 cm height) without food hosts, which was then kept at a RH of 70–75% in a humidity chamber (27 × 20 × 17 cm). To maintain the abovementioned RH, saturated salt (NaCl) solutions (Duksan Pure Chemicals, Ansan-si, Gyeonggi Province, Republic of Korea) were used as described in [56]. Before the humidity chamber was placed into an environmental chamber, each humidity chamber was affixed with a HOBO data logger (Huato Log-USB, Huato Electronic Co., Ltd., Baoan District, Shenzhen, China) to measure the actual temperature and humidity within the environmental chambers. Following this, the humidity chambers were transferred to the incubators (Eyela, model MTI-202B, Tokyo, Japan) and the temperatures were set to 15, 20, 25, 27, 30, 35, and 40 °C, and a 16:8 h L:D photoperiod. Once the eggs had hatched, the egg hatching period was recorded at different temperatures. Newly hatched larvae (first instar) were transferred to a separate Petri dish (5 cm dia. × 1.5 cm height, with topside ventilation) with one of the host foods (*n* = 37 to 58/host food) [52]. The single Petri dishes (5 cm dia. × 1.5 cm height, with topside ventilation) with an individual larva on each host plant and with an artificial diet were sealed with para-film, and then kept inside the same humidity chamber and environmental chamber. A single leaf dish for soybean, single leaf piece for maize, multiple leaves for groundnut, and single leaf for azuki bean from the middle of the plant of each host plant, along with the artificial diet, equivalent to 2.5 g, were replaced with new ones daily, and then each Petri dish was sealed with para-film. Observations on the duration of each developmental stage (egg, larva, pupa, and adult) at each temperature and host plant were carried out every 12 h (morning and evening) until adult emergence or death for each development stage. Once the adult emerged, their sex was determined based on their morphology [57]. Each sexed adult was then kept individually in Petri dishes (5 cm dia. × 1.5 cm height, with topside ventilation) for measuring adult longevity at the respective temperatures and host plants. The adults were fed 10% honey–water solution.

### 2.4. Criteria for Judging Model

Several statistical criteria have been proposed to evaluate model performance including the Pearson χ^2^, coefficient of determination (r^2^) and information criteria such as the Akaikes and Bayes-Schwarz information. We evaluated the models as following: number of fitted coefficients, biological interpretation of fitted coefficients, the Pearson χ^2^, coefficient of determination (for linear model), or the coefficient of nonlinear regression. The higher the value of r^2,^ the better the fit is [58,59].

### 2.5. Developmental Distribution Model

The Weibull function is a continuous probability distribution function that can fit an extensive range of distribution shapes. The Weibull function could be applied in a wide variety of data analysis and prediction with small samples. Cumulative probability distributions of insect development time are presented in time because insect development periods depend on temperature. The probability distribution of development times for individuals in a population is described by a cumulative Weibull function. Further, the development period of each life stage was expressed in days spent at the specific temperature, and was normalized by dividing the number of days by the mean development time of the temperature. To generate a temperature-independent distribution model for each life stage, the cumulative proportion was plotted against the normalized times. Cumulative proportion was analyzed by dividing the cumulative number of completing any life stage at a normalized time by the total number of completing any life stage. This temperature-independent distribution can be used to predict the development times of individual in a population under variable temperature conditions. The relationships between the cumulative proportion of each life stage and the developmental times [60,61], which help to predict the minor data with the Weibull function (Equation (1)), were estimated by fitting a two-parameter Weibull distribution function:

(1) $$F\left(x\right)=1-\exp(-({\frac{x}{\alpha })}^{\beta })$$

where F(x) is the cumulative proportion at development time *x*, *α* is the scale parameter, and *β* is the parameter of the curve shape.

The median development time (i.e., time to 50% cumulative frequency) was calculated as *α* × [−ln(0.5)]^1/*β*^. To generate a temperature-independent distribution model for each stage, the cumulative frequency was plotted against the normalized time, which was calculated by dividing the development time by the median development time at each temperature (i.e., development days/median days). The normalized data were pooled across temperatures and fit into the Weibull model [60].

### 2.6. Developmental Rate Models

Linear and nonlinear functions were used to describe the relationship between the developmental rates (1/developmental periods) and temperature. The lower developmental threshold (LDT,$-\frac{b}{a}$) and thermal constant (K, $\frac{1}{a}$) were estimated using a linear function (y = aT + b, where y is the developmental rate and T is the assessed temperature) [62], and the parameters of the linear equation were estimated using the TableCurve 2D program (1996).

Among the various nonlinear equations that have been proposed to describe the relationship between developmental rates and temperature [63,64,65,66,67], we selected the Sharpe–Schoolfield–Ikemoto (SSI) model (Equation (2)).


(2) $$r\left(T\right)=\frac{\rho_{\phi }\frac{T}{T_{\phi }}\exp[\frac{\Delta HA}{R}\left(\frac{1}{T_{\phi }}-\frac{1}{T}\right)]}{1+\exp\left[\frac{\Delta HL}{R}\left(\frac{1}{TL}-\frac{1}{T}\right)\right]+\exp[\frac{\Delta HH}{R}\left(\frac{1}{TH}-\frac{1}{T}\right)]}$$

The SSI model, based on thermodynamics, was proposed by [68] and subsequently modified by [69,70]. It presents the temperature-dependent reaction rates of active temperature ranges for a theoretical rate-controlling enzyme. The parameters of the SSI model were estimated using an R script (2015) developed by [71]. In Equation (2), *r*(*T*) represents the developmental rate at the absolute temperature *T* (°K); Δ*HA*, Δ*HL*, and Δ*HH* are enthalpy changes (Jmol^−1^); *R* is the universal gas constant; $\rho_{\phi }$ is the developmental rate at $T_{\phi }$; and *TL* and *TH* are the temperatures at which the rate-controlling enzyme has an equal probability of being active or inactive, depending on low- or high-temperature inactivation, respectively. $T_{\phi }$ is the intrinsic optimum temperature at which the species can optimize its fitness to the environment [68,70].


(3) $$P_{2}\left(T\right)=\frac{1}{1+\exp[\frac{∆HL}{R}(\frac{1}{TL}-\frac{1}{T})]+exp[\frac{∆HH}{R}(\frac{1}{TH}-\frac{1}{T})]}$$

Equation (3) is the probability that the rate-controlling enzyme is in the active state. The maximum probability of $P_{2}(T)$ is observed at $T_{\phi }$.

### 2.7. Simulation of Adult Emergence

The adult emergence of *S. litura* was simulated according to temperature (°C) and time (day) by incorporating two functions of *S. litura* development: the Weibull function for the development distribution, and the SSI model for the temperature-dependent rate. The SSI model was also used to measure the rate of daily emergence at a given temperature, and the cumulative frequency of the adults was calculated using the Weibull distribution model (Equation (4)):

(4) $$F\left(x,T\right)=1-\exp[-({\frac{xr\left(T\right)}{\alpha })}^{\beta }]$$

where F(x, T) is the cumulative proportion of the emergence of *S. litura* adults at time *x* and constant temperature T, *x* is the time (day), *r*(*T*) is the development rate model, and *α* and *β* are parameters from the Weibull equation.

### 2.8. Composition of Host Plant Nutrient Contents

Lipids: The lipid content was analyzed by preparing a 2 g sample of ground leaves of each host plant and automatically weighing the container containing the sample. Each sample was then kept in a fat extraction thimble (BUCHI Extraction Thimbles 25 × 100 mm), using an n-hexane solvent for an automatic maintenance extraction system (BUCHI Labotechnik, B-811, AG, Meierseggstrasse 40, Postfach, Flawil, Switzerland). The reaction was carried out at a temperature of 105 °C for 2 h and 40 min. The weight of the extracted fat was measured after sample cooling for 30 min in the desiccator [72].

Protein: The moisture content of each host plant was maintained at 12–13% by drying with a hot air dryer (40 °C), at which point they were all ground (C/11/1, Glenmills, Middletown, PA, USA). The ground leaf sample (50 g) was then wrapped in lactic acid paper and analyzed using an automatic Dumas Combustion Analyzer (Rapid N Cube, Elementar Analysensysteme GmbH, Langenselbold, Germany) [73].

Free sugar: The analysis of free sugar was initiated with grinding the leaves of each host plant to make ground leaf samples. For this, 1 g of the ground leaf sample of the host plant was placed in a Falcon tube with 10 mL of 70% EtOH and shaken for 3 h in a stirrer. Then, the sample filtration process with filter paper was conducted 24 h after the addition of EtOH. A vortex mixer was used to mix the filtered solution and H_2_O in a 1:1 (*v*:*v*) ratio, and then the free sugar content was analyzed by HPLC (Ultimate 3000; Thermo Scientific, Waltham, MA, USA) in an autosampler vial [72]. Approximately 2–3 week-old leaves of each host plant were used in the nutrient content analysis.

### 2.9. Statistical Analysis

Regression analyses were performed for modeling the temperature-dependent development of each *S. litura* stage, and the model parameter values for the linear and nonlinear functions were estimated using the TableCurve 2D Automated Curve Fitting program [74]. The effects of temperature on developmental time of each stage, % survivorship, adult longevity of *S. litura*, the nutrient content of the host plants, and the combined effect of temperature and host plant were analyzed using analysis of variance (ANOVA) PROC GLM. Tukey’s Studentized Range Honestly Significant Difference (HSD) test was used for further separation of means among the factors. The differences in adult longevity between females and males were compared with a *t*-test. The relationships of (1) lipid to larval developmental time and adult emergence rate, and (2) protein to larval development and adult emergence rate were calculated using the GLM module. The ANOVA, *t*-test, and GLM analyses were performed using the SAS program [75].

## 3. Results

### 3.1. Developmental Period

The developmental period of the eggs, larvae, pupae, and total immature stage of *S. litura* varied significantly among the temperatures, although development occurred successfully on all host plants at all temperatures except 40 °C, at which no eggs hatched (Table 1). The developmental period trend of *S. litura* was inverse with temperature from 15 to 35 °C. The mean time required for the development from egg to adult emergence ranged from: 106.29 d at 15 °C to 22.47 d at 35 °C on soybean; 107.57 d at 15 °C to 21.25 d at 35 °C on maize; 130.40 d at 15 °C to 25.31 d at 35 °C on groundnut; 111.82 d at 15 °C to 18.30 d at 35 °C on azuki bean; and 103.66 d at 15 °C to 22.50 d at 35 °C on the artificial diet.

### 3.2. Survivorship

**Figure 1:**
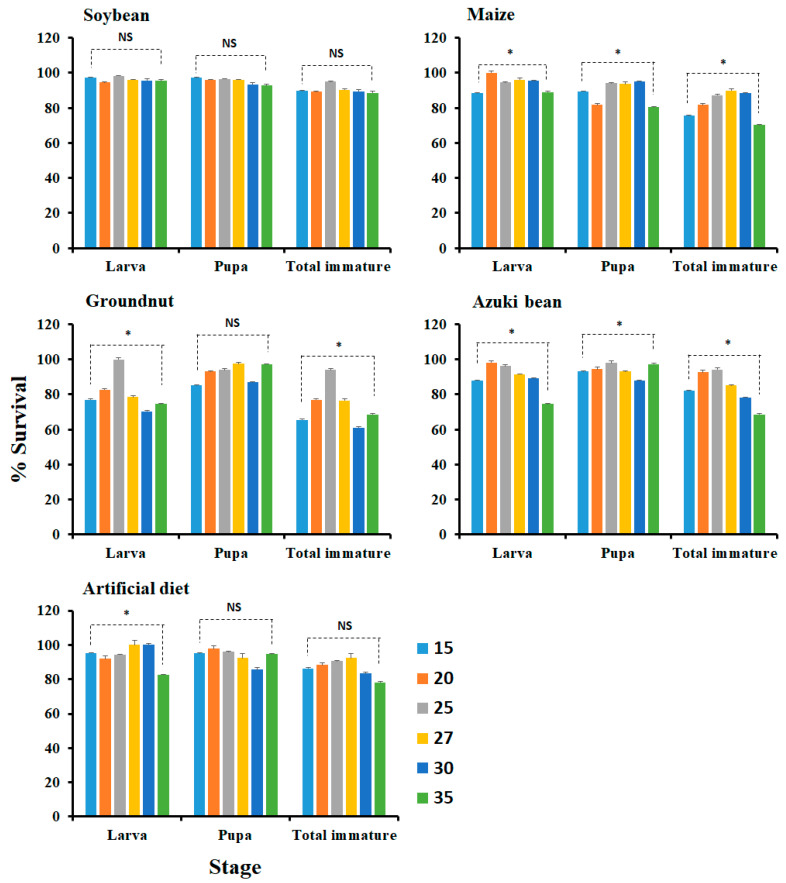
Survivorship of different stages (larva, pupa, and total immature stage) of *Spodoptera litura* at different constant temperatures (15, 20, 25, 27, 30, and 35 °C) on host plants (soybean, maize, groundnut, and azuki bean) and the artificial diet. No eggs hatched at 40 °C. Bars represent standard error and NS denotes not significant. * Significantly different among temperatures (ANOVA, Tukey’s test, *p* < 0.05).

The survival percent of each life stage at different constant temperatures is presented in Figure 1. As a result, on soybean, the survival of larva (F_5, 287_ = 0.29, *p =* 0.9189), pupal (F_5, 252_ = 0.22, *p =* 0.9523), and total immature (F_5, 291_ = 0.34, *p =* 0.8892) was not significantly different among temperatures. On maize, the survival of larva (F_5, 284_ = 4.96, *p =* 0.0002), pupa (F_5, 279_ = 3.10, *p =* 0.0009), and total immature (F_5, 289_ = 2.16, *p =* 0.0.0448) was significantly different among temperatures. On groundnut, the survival of larva (F_5, 308_ = 3.75, *p =* 0.0026) and total immature (F_5, 312_ = 3.68, *p =* 0.0030) were significantly different, while survival of pupa (F_5, 271_ = 1.29, *p =* 0.2781) was not significantly different among temperatures. On azuki bean, the survival of larva (F_5, 303_ = 2.52, *p =* 0.0294), pupal (F_5, 274_ = 3.03, *p =* 0.0111), and total immature (F_5, 303_ = 3.54, *p =* 0.0040) was significantly different among temperatures. On artificial diet, the survival of larva (F_5, 279_ = 3.57, *p =* 0.0038) was significantly different, while pupa (F_5, 262_ = 1.33, *p =* 0.2527), and total immature (F_5, 283_ = 1.08, *p =* 0.3692) was not significantly different among temperatures. On the soybean, all the stages equally survived among temperatures. On the maize, the higher percent of survival—100% of larvae, 95.1% of pupa, and 88.4% of total immature—was at 20 °C, at 30 °C, and at 30 °C, respectively; on groundnut, the higher percent of survival—100% of larvae, 97.5% of pupa, and 93.9% of total immature—was at 25 °C at 27 °C, and at 25 °C, respectively; on azuki bean, the higher percent of survival—98.2% of larvae, 98.0% of pupa, and 94.1% of total immature—was at 20 °C, at 25 °C, and at 25 °C, respectively; and on the artificial diet, the higher percent of survival—98.2% of larvae, 98.0% of pupa, and 94.1% of total immature—was 100% of larvae, 97.8% for pupa, and 92.3% of total immature at 27 and 30 °C, at 20 °C, and at 27 °C, respectively. In the meantime, the bottommost survival percent of the larvae (70.4% at 30 °C), and total immature stage (61.1% at 30 °C) were measured on groundnut.

### 3.3. Adult Longevity and Sex Ratio

In comparison, the adult longevity of *S. litura* significantly differed among the temperatures on all host plants (Table 2). The longevity between females and males also varied significantly among all temperatures for soybean and azuki bean, except at 35 °C for maize, at 30 and 27 °C for groundnut, and at 20 °C for the artificial diet. The adults were found to live longer (females: 24.89 and males: 23.96 d) at 15 °C on soybean and live shorter (males: 4.81 d) at 35 °C on groundnut. The sex ratio of almost 1:1 did not differ significantly between the moths that emerged from the host plants and those given the artificial diet (*p* > 0.05). In the meantime, the highest sex ratio (0.61) was observed at 15 °C on azuki bean, with the lowest sex ratio (0.44) at 35 °C on the artificial diet.

### 3.4. Combined Effects of Temperature and Host Plant

The development of each life stage (egg, larva, pupa, and total immature stage) was significantly impacted by both temperature and host plant (Table 3). It took longer for development at the lower extreme temperature (15 °C), whereas a shorter time was required for the development of all life stages at the upper extreme temperature (35 °C) on the host plant (*p* < 0.0001). The interaction between the temperature and host plant was also significant (*p* < 0.0001).

### 3.5. Nutrient Content

The nutrient content differed significantly among the host plants in terms of protein (F_3, 8_ = 2693.57, *p* < 0.0001), free sugar content (stachyose: F_1, 4_ = 2500.07, *p* < 0.0001; sucrose: F_3, 8_ = 738.43, *p* < 0.0001; glucose: F_3, 8_ = 467.02, *p* < 0.0001; fructose: F_1, 4_ = 9.05, *p* = 0.0396), and fatty acid compositions (C16:0-F_3, 8_ = 112.41, *p* < 0.0001; C18:0-F_3, 8_ = 153.37, *p* < 0.0001; C18:1-F_3, 8_ = 25.87, *p* = 0.0002; C18:2-F_3, 8_ = 252.63, *p* < 0.0001; C18:3-F_2, 6_ = 14.40, *p* = 0.0051; C20:0-F_2, 6_ = 20444.7, *p* < 0.0001; C20:1-F_2, 6_ = 25.10, *p* = 0.0012; C22:0-F_2, 6_ = 19.50, *p* = 0.0024). A higher level of protein (47.5%) was recorded in soybean. However, the lipid content was not significant for any of the host plants. A higher sucrose level (20.31 mg/g) was recorded in maize, followed by soybean (9.3 mg/g). However, raffinose and galactose were not detected in any of the host plants. In the case of fatty acid composition, higher levels of C16:0 (18.8%), C18:0 (5.2%), C18:2 (25.1%), and C20:0 (50.6%) were recorded in azuki bean, soybean, and groundnut, respectively (Table 4).

### 3.6. Relationship between Host Plant Nutrient Content and Life-Table Parameters

Table 5 shows the relationships between nutrient contents of host plants and life-table parameters. There were negative relationships between nutrient contents (lipid and protein) of host plant and larval development, and positive relationships between nutrient contents (lipid and protein) of host plant and adult emergence rate.

### 3.7. Developmental Distribution Model

**Figure 2:**
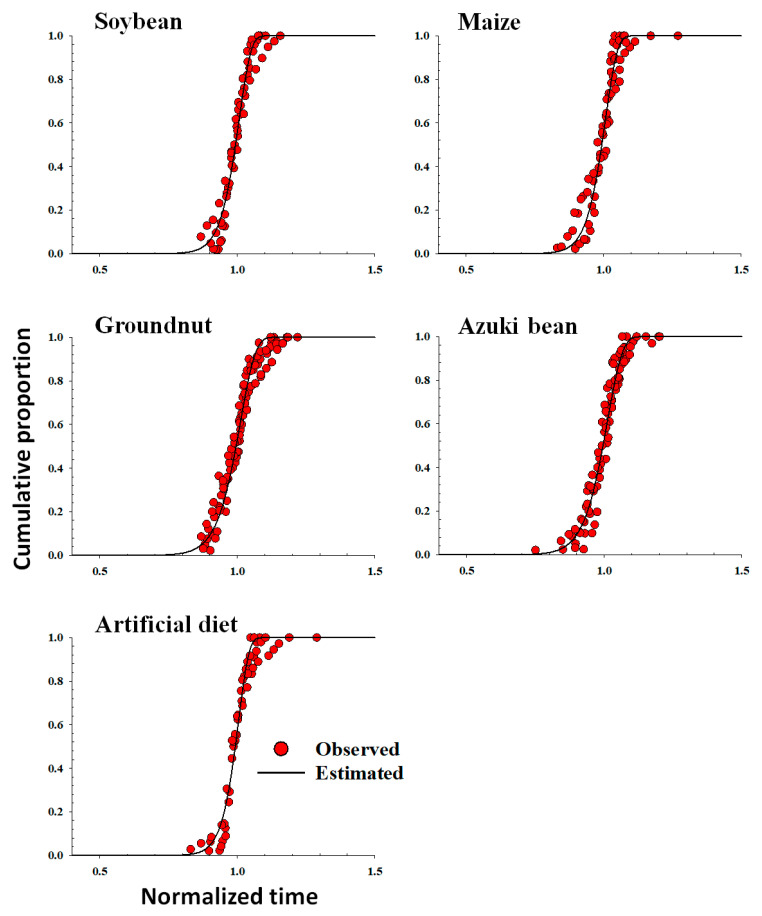
Cumulative frequency of the development of *Spodoptera litura* using host plants (soybean, maize, groundnut, and azuki bean) and the artificial diet as food sources against the normalized time (day/median day), fit to the Weibull function. Red dots: observed data; solid black lines: estimated values.

The stage-specific frequency distribution of *S. litura* against the normalized time (day/median) on host plants and artificial diet was described with the Weibull model (Table 6 and Figure 2). The cumulative frequency distribution of the developmental times of *S. litura* at each temperature on each host plant and artificial diet was found to be well fitted and described by the Weibull model (r^2^ > 0.94, *p* < 0.0001).

**Figure 3:**
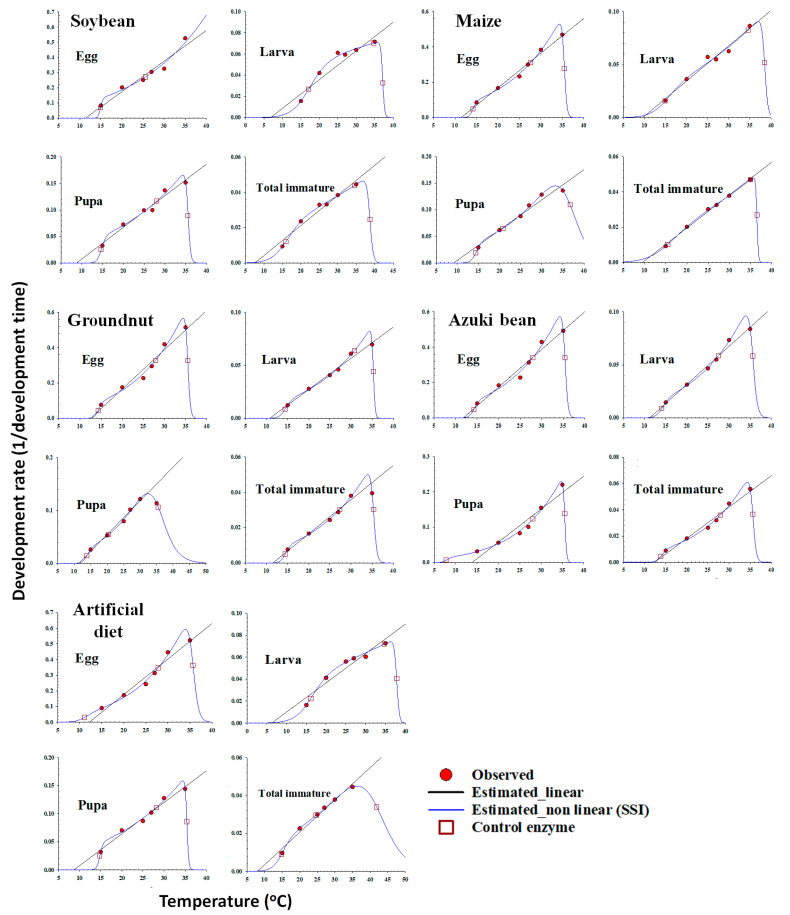
Linear, nonlinear, and enzyme control models for the temperature-dependent rate (1/day) of the development of *Spodoptera litura* using host plants (soybean, maize, groundnut, and azuki bean) and the artificial diet as food sources. Red circles: observed data; solid blue curve lines: estimated nonlinear result; solid black straight lines: linear result; red square box: control enzyme.

The relationship between temperature and the development rate of different life stages of *S. litura* was described with a linear regression model, and then this model was fit to the development rate in the mid-range on the host plants and artificial diet. The LDT and K of each life stage of *S. litura* were estimated with the regression model, and the estimates of the LDT and the K values for egg and total immature stage development were presented in Table 7 and Figure 3.

**Figure 4:**
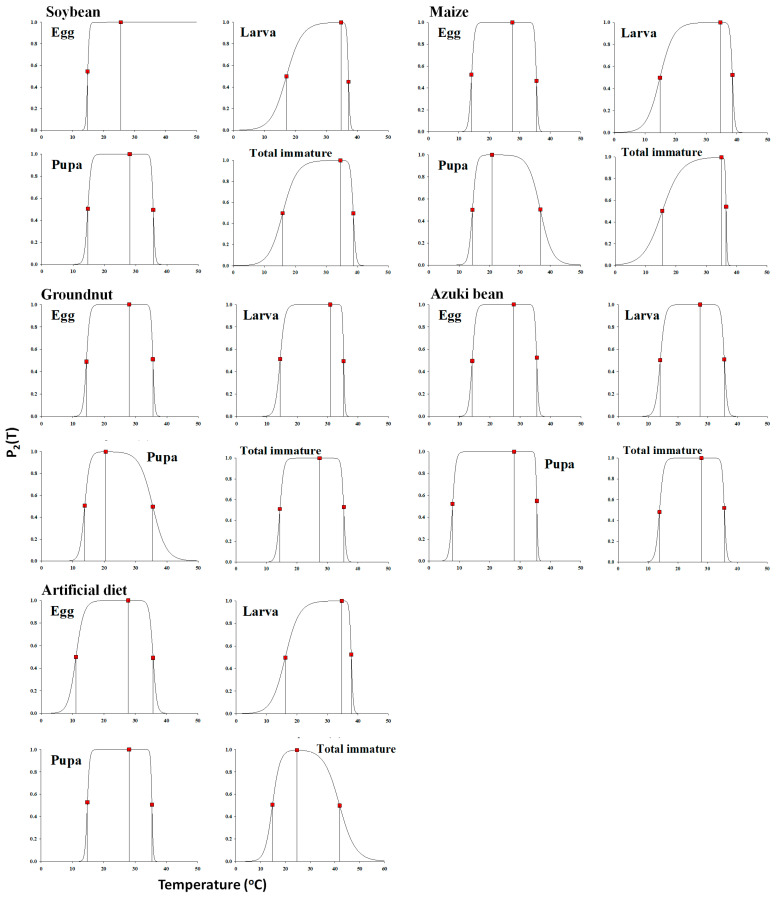
P_2_(T) is the probability that the rate-controlling enzyme of *Spodoptera litura* is in the active stage. The three closed squares from left to right represent the development rates at ${T_{L},T}_{\phi }$, and $T_{H}$. $T_{L}$ and $T_{H}$ are temperatures at which the control enzyme has equal probability to be active or inactive by low or high temperature inactivation, and $T_{\phi }$ is the intrinsic optimum temperature. Red dots: observed data; solid black curve lines: estimated data.

The estimated parameters of the nonlinear function for each life stage on each host plant are illustrated in the Table 8. A typical skewed bell shape, presenting a sharp decline when the rates were extremely high at temperatures above the optimal temperature, was noted for describing the temperature-dependent pattern of the *S. litura* development rate over the entire range (Figure 3 and Figure 4). The nonlinear model (SSI) was found as the best fit for the temperature-dependent rate of *S. litura* development on host plants and with an artificial diet (Table 4 and Figure 2). The respective intrinsic optimum temperatures (T*opt*.) of the control enzyme for the egg, larva, pupa, and total immature stage were 25.54, 34.68, 28.09, and 34.57 °C on soybean, 27.60, 34.62, 20.81, and 34.99 °C on maize, 27.96, 30.82, 20.47, and 27.39 °C on groundnut, 27.90, 34.62, 28.23, and 35.63 °C on azuki bean, and 27.77, 27.73, 14.76, and 24.65 °C on the artificial diet, respectively. The temperature window for the development of *S. litura* could be approximated from TL to TH. The temperature ranges for the total immature stage of *S. litura* reared on soybean, maize, groundnut, and the artificial diet were 22.78, 21.05, 20.98, 21.78, and 27.12 °C, respectively (Figure 4).

**Figure 5:**
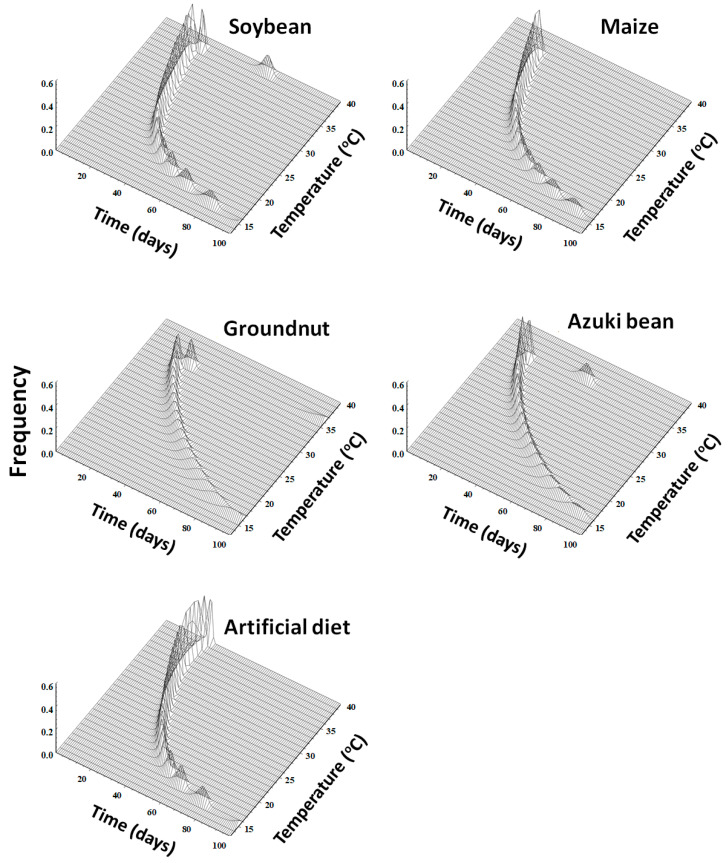
Simulation frequency of *Spodoptera litura* adult emergence from an egg cohort in relation to time (day) and constant temperature (°C) on host plants (soybean, maize, groundnut, and azuki bean) and the artificial diet.

The nonlinear function was used to predict the daily emergence of adult emergence from a cohort of *S. litura* eggs under constant temperatures, and presented in relation to temperature (°C) and time (day) (Figure 5). The adult emergence occurred earlier over a much shorter time period under optimum temperatures; however, the adult emergence exhibited a delay over extended periods under very low and very high temperatures. For example, from the cohort of eggs, adult emergence occurred between 20 and 30 days at 35 °C, whereas it took 90–97 days at 15 °C and 25–30 days at 27 °C on the host plants and the artificial diet.

### 3.8. Simulation of Adult Emergence

According to the biofix date (1 January), the voltinism of *S. litura* ranged from 4.00 to 5.47 generations on the host plants and on the artificial diet over 4 years. The number of generations was higher on soybean in 2021 and lower on groundnut in 2020. The spring emergence of adults in the field ranged from 24 May to 19 June on the host plants and on the artificial diet over 5 years. Early spring emergence (24 May) was recorded on soybean in 2022, while late spring emergence (19 June) was recorded on groundnut in 2019. However, the generation and spring emergence of adults were not significant among hosts and by year (*p* > 0.05) (Table 9).

## 4. Discussion

The surge in temperatures due to global warming has intensively impacted the biological processes of plants and insects, as well as insect population dynamics worldwide [76]. As a consequence, it is anticipated that serious problems in terms of pest management programs and challenges to food security will become more prevalent [77,78,79]. Hence, it is vital to understand the relationship between temperature and the development rate of insect pests in order to assess adult emergence in the field, develop forecasting models, and ultimately formulate integrated pest management programs [80]. Temperature is a core factor that leads to noticeable changes in the development of a wide range of insect species [81,82,83,84]. In this study, we estimated the thermal requirements of *S. litura* using linear and nonlinear developmental rate models on different host plant leaves and an artificial diet. Our study revealed that the pattern of development of *S. litura* was influenced by the studied temperatures, and the developmental pattern was inversely related to these temperatures [85,86]. Our results on the development rate of *S. litura* eggs, larvae, and pupae are largely in agreement with the studies conducted by [7,87,88], except at 15 °C. In the present study, we estimated 63.92 d on soybean and 80.20 d on groundnut at 15 °C, which are lower estimates than those found in [7,87] on soybean and groundnut, respectively, and higher estimates than those in [88] on groundnut. These variations may be due to the different host plants (especially cultivars) offered to the larvae as well as the available nutrient content and nutritional quality of the host plants [6,34,89]. The differences in chemical compounds, along with factors other than the temperature, such as insect genetic makeup, geographic origin, and environmental conditions (relative humidity and photoperiod) [90,91], emphasized that information regarding the nutritional value of the host plants offered is significant in order to develop viable and applicable management strategies against polyphagous insects, because the economic standing of polyphagous insects is mediated by the nutritional quality and by the relative abundance of the host plants. In this study, the *S. litura* eggs could not hatch or complete their development at the higher extreme temperature (40 °C) on either the host plants or the artificial diet. Previous studies have also reported the failure of egg development at extreme temperatures [42,92,93]. This abnormal development and the death of the eggs may be attributed to the rapid dehydration and physiological disorders mediated by the induced heat. Ref. [94] reported metabolic disorders and even death in insects that experienced prolonged exposure to extreme temperatures. In addition, exposure to extreme temperature regimes also inactivated the enzymes that prevent cell cycle development, thus significantly reducing the temperature range over which insect embryos develop compared to the thermal tolerance range of adults [95,96], which would explain why the extreme temperatures had such unfavorable impacts on the development of *S. litura*. However, the unsuccessful development of eggs due to extreme temperatures may vary under the fluctuating temperatures found outdoors. Ref. [97] reported that plants can maintain leaf temperatures that differ from the ambient temperature; for example, through evapotranspiration, host plants may insulate eggs from extreme temperatures. This phenomenon can be associated with egg size and proximity, and the eggs may exploit the physiology of their host’s leaves to escape from damage due to high temperatures.

In nature, the selection of a host by insect species is governed by adoptative evolution, and this selection behavior guides the evolution of host plant specificity [98,99]. Host plants play a vital role in the optimum development, mating behavior, and survival of insects in a given environmental condition, and the development and life variables of polyphagous insects are often associated with their host plants [100,101]. Within plant characteristics, the nutritional value and quality of the host plants are the significant factors that affect the survival and fitness of insects [23,25,102,103].

Insect development depends on diet to a considerable degree [25,104], and the utilization of different plant food sources brings variation to the life variables of insects [105,106]. In this study, alongside the effect of temperature (Table 3), the host plants also influenced the development and survival of *S. litura*, and the data clearly showed variation in the developmental time of the larvae, and their survival on soybean, maize, groundnut, and azuki bean. This influence of host plants on development of larvae is also backed by our regression analysis (Table 5). These findings are supported by several previous studies, despite the fact that a coordinated comparison of the findings can be difficult as diverse host plants and natural conditions were utilized in the research [6,42,51,101,107,108]. A study by [7] examining the developmental variables on soybean reported the developmental time of *S. litura* larva as 93.0 d at 15 °C, which was much longer than in this study. Other studies by [87,88] estimating the developmental variables on groundnut reported developmental times for larvae of 94.5 d and 51 d at 15 °C, respectively; the estimates of [87] were lower and those of [89] were higher than our estimates. In addition, [88] reported the developmental time of larvae on groundnut to be 17 d at 27 °C, which was approximately 4 d shorter than in this study. However, a slightly longer developmental time (23.2 d) on tobacco and shorter developmental times (15.8 and 13.3 d) on cowpea and Chinese cabbage, respectively, have been reported, in comparison to our estimates for *S. litura* larvae at 26 ± 1 °C [6]. This difference in the developmental time of *S. litura* may be due to different soybean and groundnut varieties and the nutritional quality associated with (and the quantity of) the host plant variety [98,109]. The accessibility of primary and secondary biochemicals on diverse host plants or diverse host plant parts consumed by the larvae may also have influenced the developmental time of this moth [110]. A study conducted by [111] examining the feeding environment of several insect species found that some plants contained fewer of the basic supplements necessary for the typical development of insects; specifically, insects that feed on sources with such poor nutrient levels tend to store the most important substances, such as lipids, instead of utilizing them for ordinary development, resulting in a longer development time. Other studies have also posited that this type of prolonged developmental time may be due to a lack of basic host plant nutrients [112], with nutrient deficiency ultimately leading to a longer development time in lepidopterans [113]. Refs. [114,115] reported that a lack of certain proteins may antagonistically influence the developmental times of insect species. In addition, Lepidoptera showed weak performance and slower development when they consumed leaves containing a higher amount of crude fiber and less protein [116], secondary metabolites, and toxic proteins [117,118]. We observed a longer developmental time for *S. litura* when they were fed on groundnut, which may be associated with higher amounts of secondary metabolites and toxic proteins, and less protein, as described by [118]. The influence of the nutrient contents of host plants on the developmental time of *S. litura* is well supported by the nutrient analysis carried out in this study, which clearly showed the differences in nutrient content among the host plants’ leaves, and the association between nutrient contents of host plants and life-table parameters of *S. litura* (Table 4 and Table 5).

In this study, we found that the pupal development varies among temperatures on all food hosts. In the case of pupal development, [28] reported that it was not mediated by the host plants on which the larvae fed. The developmental time of pupae on the host plants may be explained by the fact that pupal development is controlled by factors other than just the host plant, such as temperature and relative humidity, and such factors may be more critical than the host plant in some circumstances. This phenomenon is well supported by [21], who reported that temperature plays a vital role in the pupal development of *S. litura*. Further, the authors reported that the mean pupal developmental duration was as long as 13.83 d at 24 °C on perilla and as short as 7.00 d at 32 °C on soybean.

In this study, we presented evidence that temperature and host plants significantly influence the longevity of *S. litura*. However, the sex ratio (female proportion) was not biased, and almost equal numbers of female and male adults emerged when their larvae were fed with the host plants and the artificial diet. The longevities of both the female and male *S. litura* adults were also significantly affected by the host plants and the artificial diet on which their larvae fed (Table 2). The females that emerged from larvae fed with soybean lived the longest at 15 °C, while the males that emerged from larvae fed with groundnut lived the longest at 35 °C. Again, this varied outcome may be attributed to the nutritional quality and quantity of the host plant species and the biochemical components available on those plants [98,108]. We noticed significant differences between the females’ and males’ longevities among those that emerged from the host plants. These results are in agreement with the findings of previous studies [6,41,42,98]. In addition to the nutrition quality of each host plant, these longevity differences may be linked to several factors and interactions such as the complex interaction between specific native environmental conditions and sex-specific costs of reproduction, the epigenetic control of longevity by imprinting through DNA methylation, and increased fecundity and protection from aging stemming from the act of mating or components from the male ejaculate [119].

The estimation of the population development of organisms is generally carried out using origination and the development-based lower temperature threshold [120]. The estimations of the LTDs for the eggs, larvae, and pupae of *S. litura* using the linear model estimated by [7] on soybean and by [87] on groundnut are in contrast to the present study. Ref. [7] estimated lower LTDs for eggs (10.2 °C), but higher for larvae and pupae (9.9 and 9.8 °C, respectively) than we estimated in the present study. However, Ref. [87] reported lower LDTs for the egg (8.2 °C), larval (10.0 °C), and pupal (10.2 °C) stages on groundnut than our estimates. In the present study, our estimate was 45.08 DD for eggs to hatch on groundnut, which is lower than the thermal constant estimated by [87], who reported 64 DD for eggs on groundnut. However, Ref. [88] estimated lower thermal constants for larvae (303 DD) and pupae (155 DD) on groundnut than we estimated in the present study. This differences in the estimations of the lower developmental threshold and thermal constant may be attributed to differences in the rearing conditions, exposed environmental conditions, host plant variety, nutrient contents present, and the quality of the host plants used as larval food [6,42]. This reflects that there are factors other than temperature involved in the variations observed among the host plants, temperatures, and their interaction in this study (Table 3). This type of variation due to the significant interaction between temperature and diet was also noticed in other *Spodoptera* species [42,121]. It may also be due to the contrasts in each study’s exploratory technique. In this study, we allowed the larvae to pupate and remain pupae in the same Petri dish (5 cm dia. × 1.5 cm height, with topside ventilation) until the adults emerged. However, Ref. [88] used transparent plastic cups, and [7] utilized bigger Petri dishes (10 cm dia.) than we provided for our larvae, and the pupae were kept in a test tube rather than a Petri dish. It is worth considering whether the materials and techniques utilized have an influence on the developmental requirements of *S. litura*.

In the field of scientific research, there is still debate on the validity of the existing mathematical functions. Although a wide range of mathematical functions have been developed and are being used in developmental research for describing insect development rates across thermal regimes, authors tend to select models based on strong subject- and field-associated biases. Moreover, the performance of each model also varies according to the specific context, and there is no clear agreement on one particular model that can be ideally used across a wide range of applications [122]. At present, either a single model or a number of models focusing on a specific taxonomic group, frequently without justification, are used in many studies to describe temperature-dependent advancement, meaning that the key qualities of the model such as prescient control or other beneficial qualities may also be neglected [123]. Considering this shortcoming, we chose a number of diverse models to perform the data analysis. In the present study, we utilized one nonlinear numerical model (SSI) to sufficiently depict the formative rate versus the temperature curve, since the relationship between them is curvilinear near to the extremes. The reason for selecting the SSI nonlinear model is that the model offers clear biophysical meaning and thermodynamic information among the model parameters [95,124], and can best describe the temperature ranges for insect development with the temperature threshold and the maximum temperature estimates. The superior performance of the SSI nonlinear model is also supported by [125], who described that the SSI function performed as well as, or better than, other functions. In this study, we found that the high correlation coefficient (r^2^-values: 0.98–0.99) and the minimum variance of the estimated parameters on all host plants showed that the developmental rate of *S. litura* was well fitted and described using a nonlinear model. The intrinsic optimum temperature (*T*φ) value is known as the most critical factor for development that determines the fitness of an optimum life history strategy [70], which is evidence for the involvement of the maximal active state of enzymes in the development process [126,127]. The data we estimated in this study was found to fit well into the rate-control enzyme model and well described the development of *S. litura* (Figure 4).

Standard laboratory-based research conducted under a standard set of conditions, such as a consistent temperature and other controlled and replicable conditions with a specific physiological or behavioral response or survival, regularly delivers generally straightforward relationships. Nevertheless, these relationships may not be as simple as those observed under controlled conditions due to the complexity and variability experienced in field conditions, along with the involvement of little-known or unclear characteristics [128]. Ref. [129] presented evidence concerning the impact on modification within the phenotype of *Drosophila melanogaster* Meigen due to the development mediated by adult temperature acclimation. Supporting the notion of the impact of temperature acclimation, Ref. [128] detailed the versatile effects of acclimation in both laboratory and field tests, with more grounded impacts being observed within the field test. In spite of the fact that there is a difference in the relationship between the laboratory and the field, the evaluated laboratory-based traits might still be significant and pertinent to field execution, but this basic assumption should be verified [130]. Moreover, Ref. [87] reported that the response of different stages of *S. litura* to temperature under constant laboratory conditions was similar to that under field conditions.

We estimated the lower temperature threshold and thermal constant of *S. litura* using linear models because these are broadly utilized to assess the lower temperature limit and thermal constant of insect species [131] despite the fact that analysts have highlighted numerous drawbacks [132,133]. In spite of these inadequacies, linear models remain widely utilized since they require negligible information input for definitions using simple calculation. Hence, their application has generally been found to be sufficient with acceptable exactness [134]. By using a linear model with thermal constant estimates on the host plants and the artificial diet, we tested the simple application of a degree-day model with the biofix of 1 January [135,136] to predict the number of generations of *S. litura*, which resulted in 4.21–5.30 generations in 2018, 4.11–5.23 in 2019, 4.0–5.16 in 2020, 4.30–5.47 in 2021, and 4.27–5.42 in 2022 on the host plants and the artificial diet. The resulting spring emergence dates for *S. litura* were 28 May–13 June in 2018, 31 May–19 June in 2019, 26 May–13 June in 2020, 26 May–17 June in 2021, and 24 May–12 June in 2022 in the fields of the host plants in Korea (Table 8) (Maharjan, unpublished). Therefore, this study delivers important information on the temperature-dependent development of this polyphagous pest in Korea, which is anticipated to be valuable for the forecast modeling of the distribution expansion and population regulation of *S. litura* from a climate change viewpoint [133,137]. The model developed based on the thermal requirements for the development of *S. litura* estimated in this study might contribute to the advancement of integrated pest management strategies, including spray timing [138,139], with the constrained capacity of extrapolation from the laboratory-based parameter estimation [46,140]. Despite the fact that *Spodoptera* species can only successfully breed during the summer and struggle to survive the winter on the Korean peninsula due to the extreme temperatures, rapid climate change has the potential to significantly increase population, spread, and damage [141]. Global warming not only leads greater overwinter survival and an increase in population, but also impacts earlier emergence of insects in spring [142]. Based on the spring emergence trend we presented, it can be said that the emergence of *S. litura* is becoming earlier as time passes, and this may be mediated by global warming. Climate change impacts have been already experienced in the Miryang as winter temperature is getting warmer [143], and earlier appearance in spring of insects has already noticed in Miryang [144]. The spring emergence trend presented in this study is supported by our field monitoring study of *Spodoptera* species. Our field monitoring study clearly showed the early emergence and damage increment in crop fields as time passed in Miryang (Maharjan, unpublished). Further, Ref. [144] reported the earlier spring emergence of *Nysius* species (Heteroptera: Lygaeidae) in Miryang. This clearly indicates the impact of climate change on the seasonal occurrence and population dynamics of insect species in Miryang. Therefore, this climate change-mediated phenomenon could accelerate higher winter survival, lead to an increase in generations, and prolong the reproductive season, and this will ultimately help bring the great risks of profound damage of crop species and increased damage coverage of crop species, and the host shifting of insects, to both the local (Miryang) and national (Korea) levels.

## 5. Conclusions

Thermal requirements determined in this study, can be used as parameters for modeling areas and field crops that are appropriate for predicting the occurrence, number of generations, and potential distribution of *S. litura*. The optimal temperature range for egg, larval, and egg-to-adult development of *S. litura* was 25 and 30 °C. The minimum temperature threshold range for egg, larva, and pupa was 11.30 and 12.49, 6.23 and 11.19, and 8.64 and 13.78 °C on food hosts, respectively. This shows that *S. litura* will not develop and continue in those geographical regions where temperatures drop to below these temperature points during winter season. Further, in the state of changing population dynamics of insects due to climate change, we developed a model that enables correct prediction of *S. litura*, and the generated information may be a base for developing models for forecasting a wide range of insect pest species. However, the continuous monitoring of polyphagous pest species in field crops is strongly recommended.

## Figures and Tables

**Table 1:** Developmental period (day, mean ± SE) of *Spodoptera litura* on host plants (soybean, maize, groundnut, and azuki bean) and the artificial diet as food sources at a constant temperature.

Food Source	Stage	Temperature (°C)						
		40	35	30	27	25	20	15
Soybean	Egg	Dead	1.90 ± 0.1 e	3.06 ± 0.1 d	3.28 ± 0.1 d	3.97 ± 0.1 c	4.93 ± 0.1 b	12.04 ± 0.1 a
	*n*	(44)	(44)	(47)	(52)	(59)	(56)	(39)
	Larva		13.99 ± 0.2 e	15.67 ± 0.2 d	16.86 ± 0.1 c	16.36 ± 0.1 c	23.79 ± 0.1 b	63.92 ± 0.4 a
	*n*		(42)	(45)	(50)	(58)	(52)	(37)
	Pupa		6.59 ± 0.1 e	7.30 ± 0.1 d	10.02 ± 0.1 c	10.04 ± 0.1 c	13.74 ± 0.1 b	30.39 ± 0.2 a
	*n*		(39)	(42)	(48)	(56)	(50)	(36)
	Total immature stage		22.47 ± 0.3 e	26.01 ± 0.2 d	30.13 ± 0.2 c	30.38 ± 0.1 c	42.42 ± 0.2 b	106.29 ± 0.4 a
	*n*		(39)	(42)	(47)	(56)	(50)	(36)
Maize	Egg	Dead	2.13 ± 0.1 f	2.60 ± 0.2 e	3.33 ± 0.1 d	4.30 ± 0.1 c	5.96 ± 0.1 b	11.63 ± 0.1 a
	*n*	(40)	(47)	(43)	(50)	(55)	(56)	(45)
	Larva		11.56 ± 0.1 f	15.96 ± 0.2 e	18.20 ± 0.2 d	17.48 ± 0.1 c	27.51 ± 0.1 b	61.55 ± 0.4 a
	*n*		(41)	(40)	(48)	(51)	(56)	(38)
	Pupa		7.34 ± 0.2 f	7.79 ± 0.1 e	9.24 ± 0.1 d	11.38 ± 0.1 c	16.18 ± 0.1 b	34.51 ± 0.3 a
	*n*		(32)	(38)	(45)	(48)	(46)	(34)
	Total immature stage		21.25 ± 0.3 f	26.46 ± 0.3 e	30.66 ± 0.2 d	33.09 ± 0.2 c	49.57 ± 0.2 b	107.57 ± 0.6 a
	*n*		(32)	(38)	(45)	(48)	(46)	(32)
Groundnut	Egg	Dead	1.94 ± 0.1 f	2.39 ± 0.1 e	3.38 ± 0.0 d	4.30 ± 0.2 c	5.69 ± 0.1 b	13.02 ± 0.1 a
	*n*	(48)	(51)	(54)	(51)	(49)	(52)	(61)
	Larva		14.37 ± 0.4 f	16.38 ± 0.4 e	21.68 ± 0.3 d	23.19 ± 0.8 c	35.91 ± 0.5 b	80.20 ± 0.6 a
	*n*		(38)	(38)	(40)	(49)	(43)	(47)
	Pupa		8.80 ± 0.1 f	8.21 ± 0.1 e	9.83 ± 0.1 d	11.99 ± 0.4 c	18.90 ± 0.2 b	38.05 ± 0.2 a
	*n*		(37)	(33)	(39)	(46)	(40)	(40)
	Total immature stage		25.31 ± 0.4 e	26.26 ± 0.4 e	34.71 ± 0.3 d	38.91 ± 1.2 c	60.01 ± 0.7 b	130.40 ± 0.7 a
	*n*		(35)	(33)	(39)	(46)	(40)	(35)
Azuki bean	Egg	Dead	2.03 ± 0.1 f	2.31 ± 0.1 e	3.08 ± 0.1 d	4.27 ± 0.1 c	5.44 ± 0.1 b	12.22 ± 0.1 a
	*n*	(48)	(51)	(55)	(47)	(51)	(55)	(50)
	Larva		11.95 ± 0.5 f	13.41 ± 0.2 e	17.53 ± 0.6 d	20.80 ± 0.6 c	31.90 ± 0.2 b	68.40 ± 0.6 a
	*n*		(39)	(49)	(43)	(49)	(54)	(44)
	Pupa		4.41 ± 0.2 f	6.38 ± 0.1 e	9.48 ± 0.3 d	11.70 ± 0.4 c	17.72 ± 0.1 b	31.66 ± 0.4 a
	*n*		(33)	(43)	(40)	(48)	(51)	(41)
	Total immature stage		18.30 ± 0.5 f	21.94 ± 0.2 e	29.93 ± 1.0 d	36.79 ± 1.0 c	54.88 ± 0.2 b	111.82 ± 0.9 a
	*n*		(33)	(43)	(40)	(48)	(51)	(39)
Artificial diet	Egg	Dead	1.91 ± 0.1 f	2.24 ± 0.0 e	3.19 ± 0.0 d	4.11 ± 0.1 c	5.82 ± 0.1 b	11.17 ± 0.1 a
	*n*	(45)	(46)	(43)	(52)	(53)	(51)	(44)
	Larva		13.78 ± 0.4 e	16.58 ± 0.3 d	16.96 ± 0.1 d	17.88 ± 0.1 c	24.30 ± 0.2 b	61.24 ± 0.5 a
	*n*		(38)	(42)	(52)	(50)	(46)	(40)
	Pupa		6.93 ± 0.1 f	7.83 ± 0.1 e	9.80 ± 0.1 d	11.51 ± 0.1 c	14.22 ± 0.1 b	31.13 ± 0.1 a
	*n*		(36)	(36)	(48)	(48)	(45)	(38)
	Total immature stage		22.50 ± 0.5 f	26.49 ± 0.3 e	29.92 ± 0.2 d	33.44 ± 0.2 c	44.34 ± 0.2 b	103.66 ± 0.4 a
	*n*		(36)	(36)	(48)	(48)	(45)	(37)

*n*—initial no.; dead—no eggs hatched. Soybean: egg—F_5, 289_ = 911.20, *p* < 0.0001, larva—F_5, 278_ = 9450.79, *p* < 0.0001, pupa—F_5, 265_ = 5283.18, *p* < 0.0001, total immature stage—F_5, 264_ = 16,390.9, *p* < 0.0001. Maize: egg—F_5, 285_ = 1405.71, *p* < 0.0001, larva—F_5, 268_ = 8149.92, *p* < 0.0001, pupa—F_5, 237_ = 3799.85, *p* < 0.0001, total immature stage—F_5, 237_ = 9684.19, *p* < 0.0001. Groundnut: egg—F_5, 312_ = 3300.47, *p* < 0.0001, larva—F_5, 249_ = 2932.26, *p* < 0.0001, pupa—F_5, 229_ = 3668.05, *p* < 0.0001, total immature stage—F_5, 227_ = 6593.52, *p* < 0.0001. Azuki bean: egg—F_5, 303_ = 2095.22, *p* < 0.0001, larva—F_5, 272_ = 3334.13, *p* < 0.0001, pupa—F_5, 249_ = 1564.09, *p* < 0.0001; total immature stage—F_5, 250_ = 5129.82, *p* < 0.0001. Artificial diet: egg—F_5, 279_ = 1575.92, *p* < 0.0001, larva—F_5, 262_ = 3584.74, *p* < 0.0001; pupa—F_5, 245_ = 5878.05, *p* < 0.0001, total immature stage—F_5, 245_ = 9980.04, *p* < 0.0001. Means followed by the same letters in a row are not significantly different among temperatures (ANOVA, Tukey’s HSD test, *p* < 0.05).

**Table 2:** Adult longevity (day, mean ± SE) and sex ratio (female proportion) of *Spodoptera litura* reared on different host plants (soybean, maize, groundnut, azuki bean) and the artificial diet as a food source at constant temperatures.

Parameter	Food Source	Sex	Temperature (°C)						
			40	35	30	27	25	20	15
Adult longevity	Soybean	Female	Egg dead	8.80 ± 0.2 de *	8.71 ± 0.2 de *	7.77 ± 0.3 e *	9.79 ± 0.2 c *	11.72 ± 0.2 b *	24.89 ± 0.1 a *
		Male		6.64 ± 0.1 cd	6.92 ± 0.1 cd	6.43 ± 0.2 d	7.05 ± 0.1 cd	10.50 ± 0.2 b	23.96 ± 0.3 a
	Maize	Female	Egg dead	6.26 ± 0.3 e	7.23 ± 0.2 cd *	7.14 ± 0.2 de *	7.86 ± 0.2 cd *	9.24 ± 0.2 b *	17.73 ± 0.3 a *
		Male		6.13 ± 0.2 c	5.92 ± 0.1 c	6.31 ± 0.2 c	6.04 ± 0.2 c	6.97 ± 0.2 b	16.82 ± 0.4 a
	Groundnut	Female	Egg dead	6.65 ± 0.2 c *	8.02 ± 0.3 ab	8.59 ± 0.2 a	8.42 ± 0.3 ab *	7.88 ± 0.2 bc *	7.04 ± 0.2 c *
		Male		4.81 ± 0.2 c	7.46 ± 0.3 ab	8.08 ± 0.3 a	7.68 ± 0.1 ab	5.94 ± 0.2 b	4.91 ± 0.2 c
	Azuki bean	Female	Egg dead	8.52 ± 0.2 b *	9.79 ± 0.3 a *	10.38 ± 0.2 a *	9.26 ± 0.2 ab *	9.13 ± 0.2 ab *	8.14 ± 0.4 b *
		Male		6.46 ± 0.2 bc	7.33 ± 0.2 bc	9.18 ± 0.2 a	8.00 ± 0.2 ab	7.60 ± 0.2 ab	6.03 ± 0.2 c
	Artificial diet	Female	Egg dead	7.00 ± 0.3 d *	7.94 ± 0.1 cd *	8.89 ± 0.2 bc *	8.59 ± 0.1 bc *	10.36 ± 0.3 b	19.92 ± 0.2 a *
		Male		6.05 ± 0.2 d	6.94 ± 0.2 cd	7.78 ± 0.2 bc	7.50 ± 0.2 bc	10.13 ± 0.2 a	18.21 ± 0.3 b
Sex ratio	Soybean	-	-	0.46	0.50	0.51	0.52	0.50	0.54
	Maize	-	-	0.55	0.53	0.47	0.54	0.54	0.50
	Groundnut	-	-	0.54	0.55	0.56	0.46	0.55	0.58
	Azuki bean	-	-	0.52	0.51	0.53	0.48	0.45	0.61
	Artificial diet	-	-	0.44	0.47	0.48	0.46	0.51	0.54

ANOVA for soybean: female—F_5, 129_ = 102.12, *p* < 0.0001, male—F_5, 128_ = 136.06, *p* < 0.0001. Maize: female—F_5, 120_ = 73.69, *p* < 0.0001, male—F_5, 111_ = 76.03, *p* < 0.0001. Groundnut: female—F_5, 119_ = 12.56, *p* < 0.0001, male—F_5, 102_ = 48.277.00, *p* < 0.0001. Azuki bean: female—F_5, 126_ = 9.94, *p* < 0.0001, male—F_5, 117_ = 23.58, *p* < 0.0001. Artificial diet: female —F_5, 116_ = 51.80, *p* < 0.0001, male—F_5, 122_ = 50.03, *p* < 0.0001. *t*-test for soybean: 35 °C, t_1, 37_ = 6.41, *p* < 0.0001; 30 °C, t_1, 40_ = 9.17, *p* < 0.0001; 27 °C, t_1, 45_ = 3.68, *p* = 0.0006; 25 °C, t_1, 54_ = 12.06, *p* < 0.0001; 20 °C, t_1, 48_ = 4.62, *p* < 0.0001; 15 °C, t_1, 33_ = 3.77, *p* = 0.0007. Maize: 35 °C, t_1, 30_ = 0.41, *p* = 0.6862; 30 °C, t_1, 36_ = 4.71, *p* < 0.0001; 27 °C, t_1, 43_ = 3.30, *p* = 0.0019; 25 °C, t_1, 46_ = 6.23, *p* < 0.0001; 20 °C, t_1, 44_ = 8.79, *p* < 0.0001; 15 °C, t_1, 32_ = 2.35, *p* = 0.0254. Groundnut: 35 °C, t_1, 33_ = 7.59, *p* < 0.0001; 30 °C, t_1, 31_ = 1.41, *p* = 0.1675; 27 °C, t_1, 37_ = 1.64, *p* = 0.1100; 25 °C, t_1, 44_ = 2.68, *p* = 0.0102; 20 °C, t_1, 38_ = 6.62, *p* < 0.0001, 15 °C, t_1, 38_ = 7.25, *p* < 0.0001. Azuki bean: 35 °C, t_1, 30_ = 4.85, *p* < 0.0001; 30 °C, t_1, 41_ = 7.97, *p* < 0.0001; 27 °C, t_1, 38_ = 4.12, *p* = 0.0002; 25 °C, t_1, 45_ = 4.63, *p* < 0.0001; 20 °C, t_1, 49_ = 5.76, *p* < 0.0001, 15 °C, t_1, 39_ = 4.10, *p* = 0.0002. Artificial diet: 35 °C, t_1, 34_ = 3.31, *p* = 0.0022; 30 °C, t_1, 34_ = 4.42, *p* < 0.0001; 27 °C, t_1, 46_ = 4.47, *p* < 0.0001; 25 °C, t_1, 46_ = 5.29, *p* < 0.0001; 20 °C, t_1, 43_ = 0.64, *p* = 0.5272, 15 °C, t_1, 35_ = 5.03, *p* < 0.0001. Means followed by the same letters in a row are not significantly different among temperatures (ANOVA, Tukey’s HSD test, *p*< 0.05). * Significant difference between females and males (*t*-test, *p* < 0.005).

**Table 3:** ANOVA results for the combined effects of temperature and hosts (soybean, maize, groundnut, and azuki bean, and the artificial diet) and interaction of temperature and host plant on the development of egg, larva, pupa, and total immature stage of *Spodoptera litura*.

Stage	Source	DF	Sum of Squares	Mean Square	F Value	*p* > F
Egg	Model	29	16,561.532	571.087	1478.13	<0.0001
	Temperature	5	15,761.570	3152.314	8159.06	<0.0001
	Host	4	25.912	6.490	16.80	<0.0001
	Temperature × Host	20	136.62	6.831	17.68	<0.0001
Larva	Model	29	456,657.96	15,746.826	2307.77	<0.0001
	Temperature	5	422,653.94	84,530.788	12,388.4	<0.0001
	Host	4	9817.604	2454.401	359.70	<0.0001
	Temperature × Host	20	9252.333	462.616	67.80	<0.0001
Pupa	Model	29	121,627.842	4194.063	1821.91	<0.0001
	Temperature	5	90,521.636	18,104.327	7864.55	<0.0001
	Host	4	4231.41	1057.85	459.53	<0.0001
	Temperature × Host	20	24,825.38	1241.269	539.21	<0.0001
Total immature stage	Model	29	1,106,418.79	38,152.372	1583.81	<0.0001
	Temperature	5	1,058,822.23	211,764.447	8790.94	<0.0001
	Host	4	14,924.02	3731.005	154.88	<0.0001
	Temperature × Host	20	14,570.71	728.535	30.24	<0.0001

Overall model of seven temperatures (15, 20, 25, 27, 30, 35, and 40 °C) and four hosts (soybean, maize, groundnut, azuki bean), and the artificial diet on different development stages of *Spodoptera litura.*

**Table 4:** Composition of lipids (%), protein (%), free sugar content (mg/g), and fatty acid composition (%) in host plant (soybean, maize, groundnut, and azuki bean) leaves.

Nutrient Contents		Food Source (Mean ± SE)		
	Soybean	Maize	Groundnut	Azuki Bean
Lipids	4.77 ± 0.2 a	4.74 ± 0.2 a	4.59 ± 0.7 a	3.81 ± 0.3 a
Protein	47.54 ± 0.3 a	29.78 ± 0.6 b	19.06 ± 0.2 c	16.01 ± 0.1 d
Free sugar content				
Stachyose	n.d	n.d	0.39 ± 0.0 b	2.47 ± 0.0 a
Raffinose	n.d	n.d	n.d	n.d
Sucrose	9.28 ± 1.1 b	20.31 ± 0.6 a	0.46 ± 0.1 c	1.14 ± 0.0 c
Glucose	25.29 ± 1.1 a	8.54 ± 0.3 b	4.00 ± 0.0 c	2.59 ± 0.1 c
Galactose	n.d	n.d	n.d	n.d
Fructose	0.26 ± 0.0 b	0.61 ± 0.2 a	n.d	n.d
Fatty acid composition				
C16:0	14.90 ± 0.3 c	15.8 ± 0.1 b	16.23 ± 0.2 b	18.84 ± 0.2 a
C18:0	5.20 ± 0.1 a	1.70 ± 0.2 c	3.66 ± 0.1 b	3.36 ± 0.0 b
C18:1	2.90 ± 0.0 a	1.20 ± 0.0 b	3.04 ± 0.4 a	3.27 ± 0.3 a
C18:2	19.20 ± 0.6 b	19.90 ± 0.1 b	25.14 ± 0.1 a	15.45 ± 0.3 c
C18:3	56.00 ± 1.0 b	59.50 ± 0.1 a	n.d	59.08 ± 0.4 a
C20:0	0.80 ± 0.0 b	1.20 ± 0.2 b	50.58 ± 0.1 a	n.d
C20:1	0.00 ± 0.0 b	0.00 ± 0.0 b	0.25 ± 0.1 a	n.d
C22:0	0.90 ± 0.0 b	0.70 ± 0.0 c	1.10 ± 0.1 a	n.d

n.d.: not detected. Means followed by the same letters in a row are not significantly different among host plants (soybean, maize, groundnut, and azuki bean leaves) (ANOVA, Tukey’s HSD test, *p* < 0.05).

**Table 5:** Relationship between plant nutrient contents (lipids and protein) and life-table parameters (larval developmental time and adult emergence rate in host plant (soybean, maize, groundnut, and azuki bean) leaves.

Host	Nutrient Contents (%)	Parameters	Temperature (°C)											
			35		30		27		25		20		15	
			r^2^	*p*	r^2^	*p*	r^2^	*p*	r^2^	*p*	r^2^	*p*	r^2^	*p*
Soybean	Lipid	Larval developmental time	0.15	0.51	0.92	0.008	0.78	0.04	0.78	0.04	0.47	0.19	0.62	0.11
		Adult emergence rate	0.78	0.04	0.83	0.02	0.62	0.11	0.84	0.02	0.76	0.04	0.41	0.23
	Protein	Larval developmental time	0.63	0.10	0.75	0.05	0.85	0.02	0.83	0.02	0.58	0.13	0.05	0.71
		Adult emergence rate	0.86	0.02	0.88	0.01	0.84	0.02	0.82	0.03	0.37	0.47	0.06	0.77
Maize	Lipid	Larval developmental time	0.91	0.01	0.87	0.01	0.09	0.61	0.64	0.10	0.64	0.10	0.63	0.10
		Adult emergence rate	0.27	0.36	0.91	0.01	0.77	0.04	0.87	0.02	0.55	0.14	0.38	0.26
	Protein	Larval developmental time	0.06	0.67	0.79	0.04	0.82	0.03	0.78	0.04	0.06	0.67	0.08	0.62
		Adult emergence rate	0.83	0.03	0.78	0.04	0.92	0.009	0.91	0.01	0.40	0.24	0.10	0.58
Groundnut	Lipid	Larval developmental time	0.86	0.02	0.88	0.01	0.89	0.01	0.75	0.04	0.20	0.44	0.71	0.07
		Adult emergence rate	0.917	0.02	0.88	0.02	0.89	0.005	0.51	0.17	0.88	0.01	0.94	0.001
	Protein	Larval developmental time	0.72	0.05	0.79	0.04	0.81	0.03	0.77	0.04	0.52	0.16	0.31	0.32
		Adult emergence rate	0.84	0.02	0.93	0.004	0.08	0.62	0.89	0.01	0.46	0.20	0.86	0.02
Azuki bean	Lipid	Larval developmental time	0.71	0.07	0.88	0.01	0.86	0.02	0.13	0.55	0.23	0.41	0.78	0.04
		Adult emergence rate	0.31	0.32	0.80	0.03	0.90	0.008	0.73	0.05	0.70	0.07	0.80	0.04
	Protein	Larval developmental time	0.32	0.31	0.79	0.04	0.74	0.05	0.66	0.09	0.24	0.39	0.44	0.21
		Adult emergence rate	0.94	0.002	0.89	0.01	0.74	0.05	0.94	0.005	0.95	0.004	0.89	0.01

Regression, Significant at *p* < 0.05.

**Table 6:** Parameter estimates (mean ± SE) of the Weibull distribution model for the development of *Spodoptera litura* using host plants (soybean, maize, groundnut, and azuki bean), and on the artificial diet as food sources against the normalized time (day/mean).

Food Source	Parameters	Estimate ± SE	*r* ^2^
Soybean	*α*	1.0081 ± 0.0014	0.96
	*β*	26.2859 ± 1.3819	
Maize	*α*	1.0122 ± 0.0015	0.94
	*β*	24.8533 ± 1.3251	
Ground nut	*α*	1.0154 ± 0.0016	0.96
	*β*	16.3904 ± 0.6296	
Azuki bean	*α*	1.0147 ± 0.0016	0.96
	*β*	19.4899 ± 0.8541	
Artificial diet	*α*	1.0065 ± 0.0014	0.95
	*β*	30.2039 ± 1.8799	

Soybean: F_1, 65_ = 1403.15, *p* < 0.0001. Maize: F_1, 79_ = 1236.95, *p* < 0.0001. Groundnut: F_1, 93_ = 2238.66, *p* < 0.0001. Azuki bean: F_1, 84_ = 1833.94, *p* < 0.0001. Artificial diet: F_1, 61_ = 1125.56, *p* < 0.0001.

**Table 7:** Linear regression analysis for *Spodoptera litura* using host plants (soybean, maize, groundnut, and azuki bean) and the artificial diet as food sources.

Food Source	Life Stage	Linear Regression	LDT	K (DD)
Soybean	Egg	−0.2274 + 0.0201 T	11.30	49.67
	Larva	−0.0182 + 0.0028 T	6.75	369.58
	Pupa	−0.0521 + 0.0059 T	8.74	167.62
	Total immature stage	−0.0127 + 0.0017 T	7.50	587.88
Maize	Egg	−0.2235 + 0.0196 T	11.40	51.00
	Larva	−0.0316 + 0.0033 T	9.54	301.85
	Pupa	−0.0519 + 0.0057 T	9.14	176.23
	Total immature stage	−0.0176 + 0.0018 T	9.48	536.84
Groundnut	Egg	−0.2769 + 0.0222 T	12.49	45.08
	Larva	−0.0314 + 0.0029 T	10.70	340.74
	Pupa	−0.0721 + 0.0063 T	11.34	157.49
	Total immature stage	−0.0221 + 0.0019 T	11.44	517.45
Azuki bean	Egg	−0.2505 + 0.0212 T	11.78	47.02
	Larva	−0.0402 + 0.0035 T	11.19	278.59
	Pupa	−0.1285 + 0.0093 T	13.78	107.25
	Total immature stage	−0.0293 + 0.0023 T	12.32	419.44
Artificial diet	Egg	−0.2759 + 0.0227 T	12.18	44.16
	Larva	−0.0166 + 0.0027 T	6.23	375.57
	Pupa	−0.0486 + 0.0056 T	8.64	177.82
	Total immature stage	−0.0135 + 0.0017 T	7.95	586.95

LDT: lower developmental threshold; K: thermal constant; and DD: degree-day. Soybean: egg F_1, 4_ = 62.76, *p* < 0.0013, r^2^ = 0.94, larva F_1, 3_ = 43.24, *p* < 0.0072, r^2^ = 0.94, pupa F_1, 4_ = 133.89, *p* < 0.0003, r^2^ = 0.97, total immature stage F_1, 4_ = 92.35, *p* < 0.0007, r^2^ = 0.96. Maize: egg F_1, 4_ = 214.19, *p* < 0.0001, r^2^ = 0.98, larva F_1, 4_ = 136.43, *p* < 0.0003, r^2^ = 0.97, pupa F_1, 4_ = 116.01, *p* < 0.0004, r^2^ = 0.97, total immature stage F_1, 4_ = 1024.23, *p* < 0.0001, r^2^ = 0.99. Groundnut: egg F_1, 4_ = 103.04, *p* < 0.0005, r^2^ = 0.96, larva F_1, 4_ = 317.42, *p* < 0.0001, r^2^ = 0.99, pupa F_1, 3_ = 214.66, *p* < 0.0007, r^2^ = 0.99, total immature stage F_1, 3_ = 149.91, *p* < 0.0011, r^2^ = 0.98. Azuki bean: egg F_1, 4_ = 92.49, *p* < 0.0007, r^2^ = 0.96, larva F_1, 4_ = 258.68, *p* < 0.0001, r^2^ = 0.99, pupa F_1, 4_ = 46.53, *p* < 0.0024, r^2^ = 0.92, total immature stage F_1, 4_ = 141.83, *p* < 0.0003, r^2^ = 0.97. Artificial diet: egg F_1, 4_ = 97.66, *p* < 0.0006, r^2^ = 0.96, larva F_1, 4_ = 50.17, *p* < 0.0021, r^2^ = 0.93, pupa F_1, 4_ = 207.00, *p* < 0.0001, r^2^ = 0.98, total immature stage F_1, 4_ = 208.90, *p* < 0.0001, r^2^ = 0.98.

**Table 8:** Parameter estimates of the nonlinear developmental rate model for *Spodoptera litura* using host plants (soybean, maize, groundnut, and azuki bean) and the artificial diet as food sources.

Food Source	Function	Temperature (°C)	Life Stage			
			Egg	Larva	Pupa	Total Immature Stage
Soybean	SSI	*Ρ_Φ_*	0.2716	0.0709	0.1169	0.0439
		*Τ_Φ_*	298.6939	307.8292	301.2384	307.7191
		Δ*HA*	11,199.21	3867.898	10,192.98	5063.22
		Δ*HL*	−594,857	−73,021.98	−302,283.8	−72,736.21
		Δ*HH*	75,041.44	587,556.1	595,552.3	437,736.2
		*TL*	287.9996	289.2787	287.8419	289.0628
		*TH*	413.6936	310.9827	308.7422	311.8452
		*χ* ^2^	0.0032	0.0002	0.0009	0.0001
		*r^2^*	0.98	0.99	0.99	0.99
Maize	SSI	*Ρ_Φ_*	0.3102	0.0827	0.0651	0.0471
		*Τ_Φ_*	300.7526	307.7726	293.9558	308.1407
		Δ*HA*	14,653.13	8022.204	13,116.65	6917.36
		Δ*HL*	−330,938.4	−74,671.68	−247,846.3	−53,373.57
		Δ*HH*	623,290.7	499,347.5	96,785.11	1,100,002
		*TL*	287.3062	288.0615	287.5453	288.6234
		*TH*	308.6088	311.6873	309.8889	309.6783
		*χ* ^2^	0.0008	0.001	0.0001	0.00002
		*r^2^*	0.99	0.99	0.99	0.99
Groundnut	SSI	*Ρ_Φ_*	0.3313	0.0639	0.0544	0.0304
		*Τ_Φ_*	301.1109	303.9689	293.6146	300.5481
		Δ*HA*	15,535.72	13,311.59	15,759.67	14,173.82
		Δ*HL*	−305,494.2	−205,402.2	−204,729.6	−262,107.3
		Δ*HH*	609,146	870,234.4	87,953.03	474,535.6
		*TL*	287.4733	287.5099	286.9245	287.5212
		*TH*	308.6638	308.4457	308.6361	308.4988
		*χ* ^2^	0.0028	0.00009	0.0002	0.00006
		*r^2^*	0.99	0.99	0.99	0.99
Azuki bean	SSI	*Ρ_Φ_*	0.3409	0.0587	0.1207	0.0359
		*Τ_Φ_*	301.0599	308.7712	301.2314	308.7823
		Δ*HA*	15,333.69	14,564.57	17,443.35	15,634.07
		Δ*HL*	−277,185.7	−197,636.9	−283,992.3	−235,617.8
		Δ*HH*	559,231.3	365,281.5	878,494.5	479,599.9
		*TL*	287.3601	287.2409	280.8971	287.0005
		*TH*	308.5834	308.7712	308.6929	308.7823
		*χ* ^2^	0.0038	0.00008	0.0014	0.0003
		*r^2^*	0.98	0.99	0.99	0.99
Artificial diet	SSI	*Ρ_Φ_*	0.3465	0.3447	0.1107	0.0296
		*Τ_Φ_*	300.9194	300.8777	287.9077	297.8031
		Δ*HA*	17,010.89	16,933.11	10,188.86	8357.665
		Δ*HL*	−127,821.2	−234,056.1	−443,479.4	−108,487.9
		Δ*HH*	313,554.3	501,103.8	885,759.7	62,185.09
		*TL*	284.2495	286.1382	287.9077	287.9056
		*TH*	308.831	308.5825	308.5554	315.0288
		*χ* ^2^	0.0018	0.0018	0.0004	0.000005
		*r^2^*	0.99	0.99	0.99	0.99

**Table 9:** Estimated annual voltinism over five-year period in Miryang, Republic of Korea, based on a biofix of 1 January, and date of spring emergence of *Spodoptera litura* adults.

Year	Food Source	Biofix of 1 January		
		Thermal Constant (DD)	No. of Generations	Emergence Date
2018	Soybean	600.75	5.30	28 May
	Maize	547.43	4.90	3 June
	Groundnut	517.89	4.21	13 June
	Azuki bean	425.89	4.74	10 June
	Artificial diet	600.35	5.11	30 May
2019	Soybean	598.40	5.23	31 May
	Maize	541.92	4.81	8 June
	Groundnut	530.10	4.11	19 June
	Azuki bean	420.86	4.62	15 June
	Artificial diet	587.15	5.04	2 June
2020	Soybean	590.35	5.16	26 May
	Maize	546.87	4.70	4 June
	Groundnut	530.92	4.00	13 June
	Azuki bean	431.37	4.50	10 June
	Artificial diet	588.70	4.96	29 May
2021	Soybean	589.50	5.47	26 May
	Maize	545.57	5.02	6 June
	Groundnut	524.59	4.30	17 June
	Azuki bean	425.58	4.85	15 June
	Artificial diet	594.45	5.27	30 May
2022	Soybean	587.00	5.42	24 May
	Maize	540.88	4.99	31 May
	Groundnut	519.84	4.27	12 June
	Azuki bean	423.26	4.80	9 June
	Artificial diet	598.30	5.23	27 May

Spring emergence dates for *S. litura* adults were predicted from the degree-day calculation from the same weather data of each year for all host plants (soybean, maize, groundnut, and azuki bean) and the artificial diet, based on the lower development threshold.

## Data Availability

Data are available upon request to the corresponding author.
